# Identification of modifiable pre- and postnatal dietary and environmental exposures associated with owner-reported canine atopic dermatitis in Finland using a web-based questionnaire

**DOI:** 10.1371/journal.pone.0225675

**Published:** 2020-05-29

**Authors:** Manal Hemida, Kristiina A. Vuori, Siru Salin, Robin Moore, Johanna Anturaniemi, Anna Hielm-Björkman

**Affiliations:** 1 Department of Equine and Small Animal Medicine, Faculty of Veterinary Medicine, University of Helsinki, Helsinki, Finland; 2 Department of Nutrition and Clinical Nutrition, Faculty of Veterinary Medicine, Beni-Suef University, Beni-Suef, Egypt; Faculty of Animal Sciences and Food Engineering, University of São Paulo, BRAZIL

## Abstract

A cross-sectional hypothesis generating study was performed to investigate modifiable exposures such as whether feeding pattern (a non-processed meat based diet, NPMD, or an ultra-processed carbohydrate based diet, UPCD), certain environmental factors and their timing of exposure might be associated with the development of canine atopic dermatitis (CAD). Also, genetic and demographic factors were tested for associations with CAD. The data was collected from the validated internet-based DogRisk food frequency questionnaire in Finland. A total of 2236 dogs were eligible for the study (the owners reported 406 cases and 1830 controls). Our main interest was to analyze modifiable early risk factors of CAD, focusing on nutritional and environmental factors. We tested four early life periods; prenatal, neonatal, early postnatal and late postnatal periods. Twenty-two variables were tested for associations with CAD using logistic regression analysis. From the final models we identified novel dietary associations with CAD: the NPMD during the prenatal and early postnatal periods had a significant negative association with the incidence of CAD in adult dogs (age above 1 year). Oppositely, UPCD was associated with a significantly higher risk for CAD incidence. Other variables that were associated with a significantly lower risk for CAD were maternal deworming during pregnancy, sunlight exposure during early postnatal period, normal body condition score during the early postnatal period, the puppy being born within the same family that it would stay in, and spending time on a dirt or grass surface from 2 to 6 months. Also, the genetic factors regarding maternal history of CAD, allergy-prone breeds and more than 50% white-colored coat all showed a significant positive association with CAD incidence in agreement with previous findings. Although no causality can be established, feeding NPMD early in life seemed to be protective against CAD, while UPCD could be considered a risk factor. Prospective intervention studies are needed to establish the causal effects of the protective role of NPMD on prevalence of CAD during the fetal and early postnatal life.

## Introduction

Canine atopic dermatitis (CAD) is considered an incurable inflammatory and pruritic allergic skin disease in dogs, mostly diagnosed based on clinical skin symptoms and to see if it is food-induced, the clinician uses the results from an elimination diet [1, 2]. The disease prevalence is up to 10% in the dog population [3] with usual eruption within the first three years of age [1, 4]. Atopic dermatitis (AD) in humans and CAD in dogs are complex multifactorial diseases resulting from an interaction between genetics, epigenetics, immune system and environmental exposures including diet [5–8].

The genetic component and heritability of CAD has been confirmed by several genomic studies which have suggested several genes to be important in the pathogenesis [9–11]. Other studies support the heritability of CAD, for example, 50% of dogs with a paternal history of atopy develop CAD themselves [12], and the maternal history of CAD greatly increase the risk of CAD incidence in offspring [13]. A strong breed predilection to develop CAD has also been confirmed in several studies [13–16]. The most frequently affected breeds are West Highland White Terrier, Boxer, English bulldog, Dalmatian, Golden Retriever, French Bulldog, Bull Terrier, German Shepherd Dog, and English springer spaniel [7, 13, 14]. Gender plays a part in human AD predisposition, [17] and this is the same with CAD in dogs [18]. Some studies have also suggested an association between the expression of genes responsible for coat color and CAD in dogs [19–22]. Studying the genetic and background factors along with the modifiable early life factors will help us understand the etiopathogenesis of CAD and to provide ways of preventing the disease.

There is growing evidence from human epidemiological studies that early exposures during pregnancy and postnatal life are crucial for the programming of the immune system, and therefore predisposition to allergy later in life [23–29]. Early life is a critical time window when one can influence allergy risk and/or prevention. Potential risk factors for human AD and/or allergy that might have an impact on the early immune development include early life diet [30–32] and environmental exposures [33, 34], which are regarded as modifiable, whenever they affect the risk of the disease. In human studies, early nutrition and lifestyle factors have been shown to be key factors in programming for health during the critical periods, from antenatal life to early postnatal life, resulting in long-term changes related to later health conditions [29, 35, 36]. The mechanisms by which this health related programming occur is unclear, some proposed mechanisms include genetics [37], epigenetics [38] and intergenerational effects [39].

As in humans, recent studies have shown that maternal diet during lactation has an effect on the development of allergies in canines [40, 41]. The impact of the diet on human and canine health is not only tied to their macro- or micronutrient content but also to the processing; whether the food items are consumed as raw / non-processed or as ultra-processed diets, or mixes of them, as described in the NOVA guidelines [42–45]. Wild canids naturally consume diets high in raw animal protein and fat and low in carbohydrates [46–49]. In contrast, pets are usually served ultra-processed foods with a high carbohydrate content. The so-called “Developmental Origins of Health and Disease” (DOHaD) hypothesis is the current term of the ‘Fetal Origins of Adult Disease’ concept that was established for humans in the 1990s [26], and it presumes that exposure to certain environmental stimuli during critical developmental periods may have significant impacts on an individual’s future health [27]. The evidence come from animal models and human studies [29]. As far as we know, the DOHaD hypothesis has not been tested on dogs a priori, before.

Two hypotheses were tested in our study, the developmental DOHaD hypothesis, which emphasizes the role of early life nutrition on allergy susceptibility in adult life [50], and the hygiene hypothesis which speculates that early exposure to dietary and environmental microbiota stimulates the early immune system development [51]. The influence of the maternal diet during pregnancy on prevalence of CAD has so far been poorly investigated. Moreover, the possible effects of a non-processed meat based diet (NPMD) or an ultra-processed carbohydrate based diet (UPCD) on CAD prevalence has not been tested previously. The aim of this study was to investigate whether a NPMD or an UPCD feeding pattern as well as environmental factors and their timing of exposure (in the prenatal, neonatal and postnatal life) may be associated with the development of CAD in adult dogs. We additionally aimed to test the already known genetic and demographic risk factors for CAD, in part to support them and in part to thereby validate our new results.

## Materials and methods

### Study design and data collection

This is a cross-sectional epidemiological study with longitudinal data. The data was a subset from the data of the large validated internet-based DogRisk food frequency questionnaire (FFQ) [52]. The DogRisk FFQ as described in a previous study [13] was launched in 2009 at the Faculty of Veterinary Medicine, University of Helsinki, Finland. All in all, it generates 1332 data points per dog. The questions are answered by the owners and include different aspects of nutritional and environmental exposures during the whole life of the dog, starting from prenatal life and depending on the dog’s age at time of answering, often until adult age. Besides that, there are some questions on the essential demographic information for the dogs and their owners. Moreover, the FFQ contains two question sets about the diseases of the dogs in question and those of their mothers; each of them covers 117 different canine diseases. All questions and answers given have been written using more layman terms that professional veterinary or epidemiological terms, as the FFQ was answered by laymen, e.g. “very chubby” instead of “obese”. The DogRisk FFQ is still open online http://www.ruokintakysely.fi/ and is so far available only in Finnish. The ethical approval for the questionnaire (29.4.2016) was applied from the University of Helsinki, Viikki campus ethical board.

**Fig 1:**
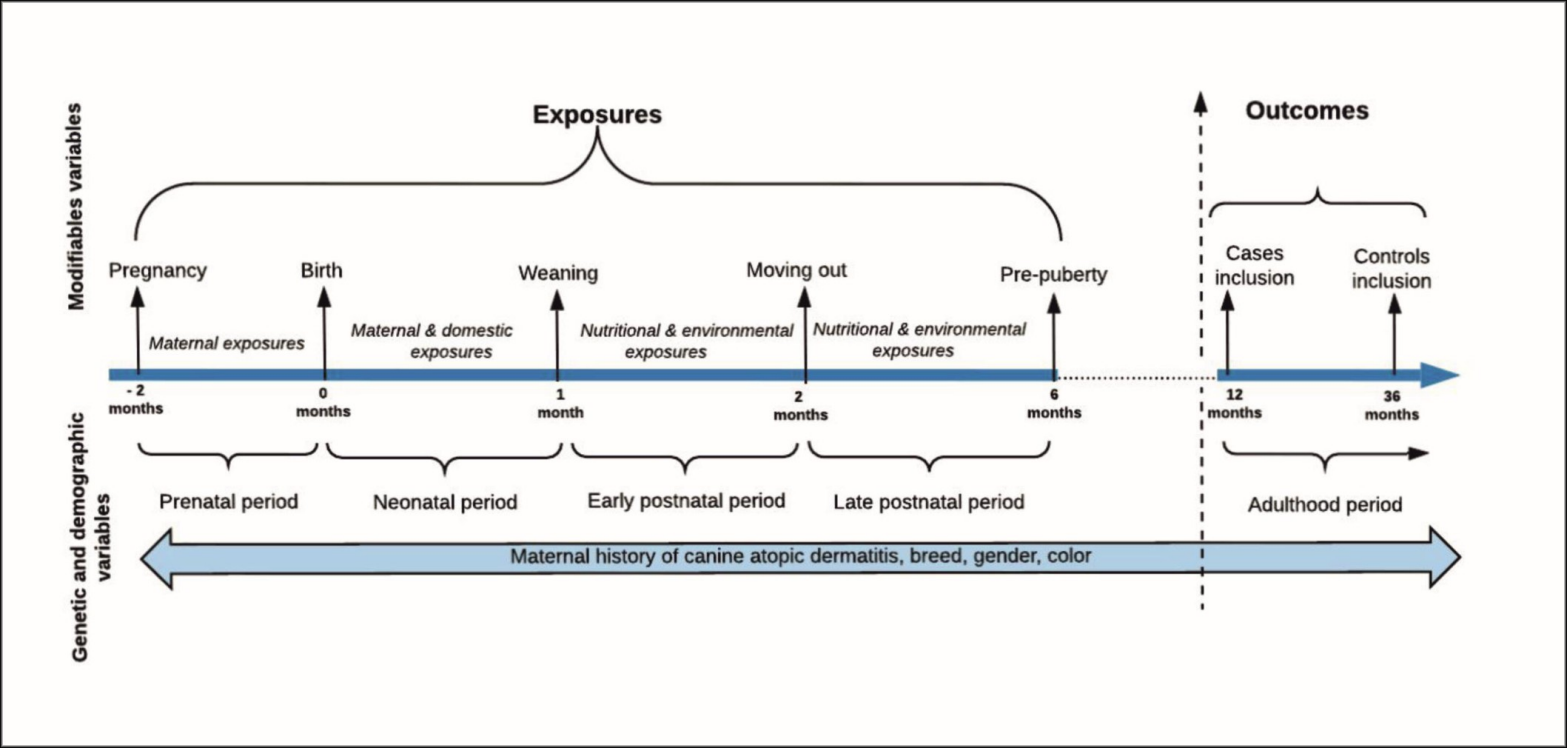
Timeline of the study design.

This binary outcome (dependent) variable was tested for any potential associations with 22 different categorical and ordinal variables (listed in Table 1) at different time points in the dog’s early life. The early life periods studied here (Fig 1) are as follow: 1) The prenatal period is the period of fetal life or intrauterine life during pregnancy. 2) The neonatal period is the period directly after birth and extends until the first 3 to 4 weeks of the puppy’s life. 3) The early postnatal period for the young puppy is the period from one to two months of age. 4) The late postnatal period for older puppies, is the period from 2–6 months of age.

**Table 1:** Variable characteristics and their distribution within study cases, controls and the total study population.

N.	Covariates	Categories	Canine atopic dermatitis %(n)		
			Cases, % (n = 406)	Controls, % (n = 1830)	Total,% (n = 2236)
**Prenatal period**					
**1**	Maternal history of CAD	Non-atopic mothers	80.1 (113)	97.8 (881)	95.4 (994)
		Atopic mothers	19.9 (28)	2.2 (20)	4.6 (48)
**2**	Dog breed	Non-allergy prone breeds	40.2 (137)	65.4 (952)	60.6 (1089)
		Allergy prone breeds	59.8 (204)	34.6 (503)	39.4 (707)
**3**	Dog gender	Male	49.4 (195)	42.9 (765)	44.1(960)
		Female	50.6 (200)	57.1 (1017)	55.9 (1217)
**4**	Dog color	White >90%	12.9 (49)	7.9 (136)	8.8 (185)
		White >50%	14.5 (55)	10.7 (184)	11.4 (239)
		Less white	21.1 (80)	26.8 (461)	25.8 (541)
		Little or no white	51.5 (195)	54.5 (936)	54.0 (1131)
**5**	Mother’s diet during pregnancy	NPMB	3.9 (10)	8.3 (94)	7.5 (104)
		UPCD	96.1 (244)	91.7 (1041)	92.5 (1285)
**6**	Was the mother dewormed during pregnancy?	Yes	94.8 (221)	97.7 (1135)	97.2 (1356)
		No	5.2 (12)	2.3 (27)	2.8 (39)
**7**	Was mother vaccinated during pregnancy?	Yes	50.4 (63)	48.5 (329)	48.8 (392)
		No	49.6 (62)	51.5 (349)	51.2 (411)
**Neonatal period**					
**8**	Mother’s diet during lactation	NPMB	5.4 (13)	7.9 (86)	7.4 (99)
		UPCD	94.6 (227)	92.1 (1004)	92.6 (1231)
**9**	Season of birth	Winter (Dec-Feb)	26.5 (106)	25.5 (460)	25.7 (566)
		Spring (March-May)	33.3 (133)	32.0 (576)	32.2 (709)
		Summer (June-Aug)	21.3 (85)	23.4 (422)	23.0 (507)
		Autumn (Sept-Nov)	19.0 (76)	19.1 (344)	19.1 (420)
**Early postnatal period**					
**10**	Puppy’s first solid diet	NPMD	3.3 (8)	8.8 (99)	7.9 (107)
		UPCD	96.7 (233)	91.2 (1020)	92.1 (1253)
**11**	Frequency of outdoor activity	Many times / day	51.1 (157)	60.0 (843)	58.4 (1000)
		Once / day	16.6 (51)	15.3 (215)	15.5 (266)
		A few times / week	12.1 (37)	12.2 (172)	12.2 (209)
		A few times / month	9.1 (28)	4.2 (59)	5.1 (87)
		Not at all	11.1 (34)	8.3 (117)	8.8 (151)
**12**	Sunlight exposure, hours / day	Not at all	20.9 (44)	14.2 (143)	15.4 (187)
		≥ 1 hour	79.1 (167)	85.8 (861)	84.6 (1028)
**13**	Type of flooring	Dirt / lawn floor	5.3 (17)	7.0 (102)	6.7 (119)
		Non-dirt / lawn floor	94.7 (301)	93.0 (1355)	93.3 (1656)
**14**	Body condition score	Overweight puppies	17.1 (60)	13.8 (213)	14.5 (273)
		Normal weight puppies	72.0 (252)	77.2 (1188)	76.3 (1440)
		Underweight puppies	10.9 (38)	8.9 (137)	9.3 (175)
**Late postnatal period**					
**15**	Puppy diet	NPMD	16.7 (41)	23.7 (247)	22.4 (288)
		UPCD	83.3 (205)	76.3 (794)	77.6 (999)
**16**	Was the dog born into the same human family as where it stayed as adult?	No	97.3 (395)	90.4 (1654)	91.6 (2049)
		Yes	2.7 (11)	9.6 (176)	8.4 (187)
**17**	Outdoor activity, hours / day	< 0.5	2.4 (8)	2.3 (35)	2.3 (43)
		0.5–1.0	32.3 (108)	26.1 (397)	27.3 (505)
		1.0–2.0	50.6 (169)	51 (775)	50.9 (944)
		> 2.0	14.7 (49)	20.5 (312)	19.5 (361)
**18**	Sunlight exposure, hours / day	≤ 1	30.8 (95)	25.5 (348)	26.5 (443)
		> 1	69.2 (213)	74.5 (1018)	73.5 (1231)
**19**	Type of flooring	Dirt / lawn	7.1 (29)	11.8 (216)	11.0 (245)
		Non-dirt / lawn	92.9 (377)	88.2 (1614)	89.0 (1991)
**20**	Body condition score	Overweight puppies	6.3 (22)	6.0 (95)	6.1 (117)
		Normal weight puppies	67.6 (236)	68.5 (1083)	68.3 (1319)
		Underweight puppies	26.1 (91)	25.5 (403)	25.6 (494)
**21**	Was the puppy vaccinated 2–4 times under 1 year of age?	Yes	99.3 (400)	99.1 (1786)	99.1 (2186)
		No	0.7 (3)	0.9 (16)	0.9 (19)
**22**	Was the puppy dewormed 2–10 times under 1 year of age?	Yes	98.7 (387)	99.3 (1760)	99.2 (2147)
		No	1.3 (5)	0.7 (12)	0.8 (17)

(n): number of dogs, CAD: canine atopic dermatitis, NPMD: non-processed meat based diet, UPCD: ultra-processed carbohydrate based diet.

### Study population

**Fig 2:**
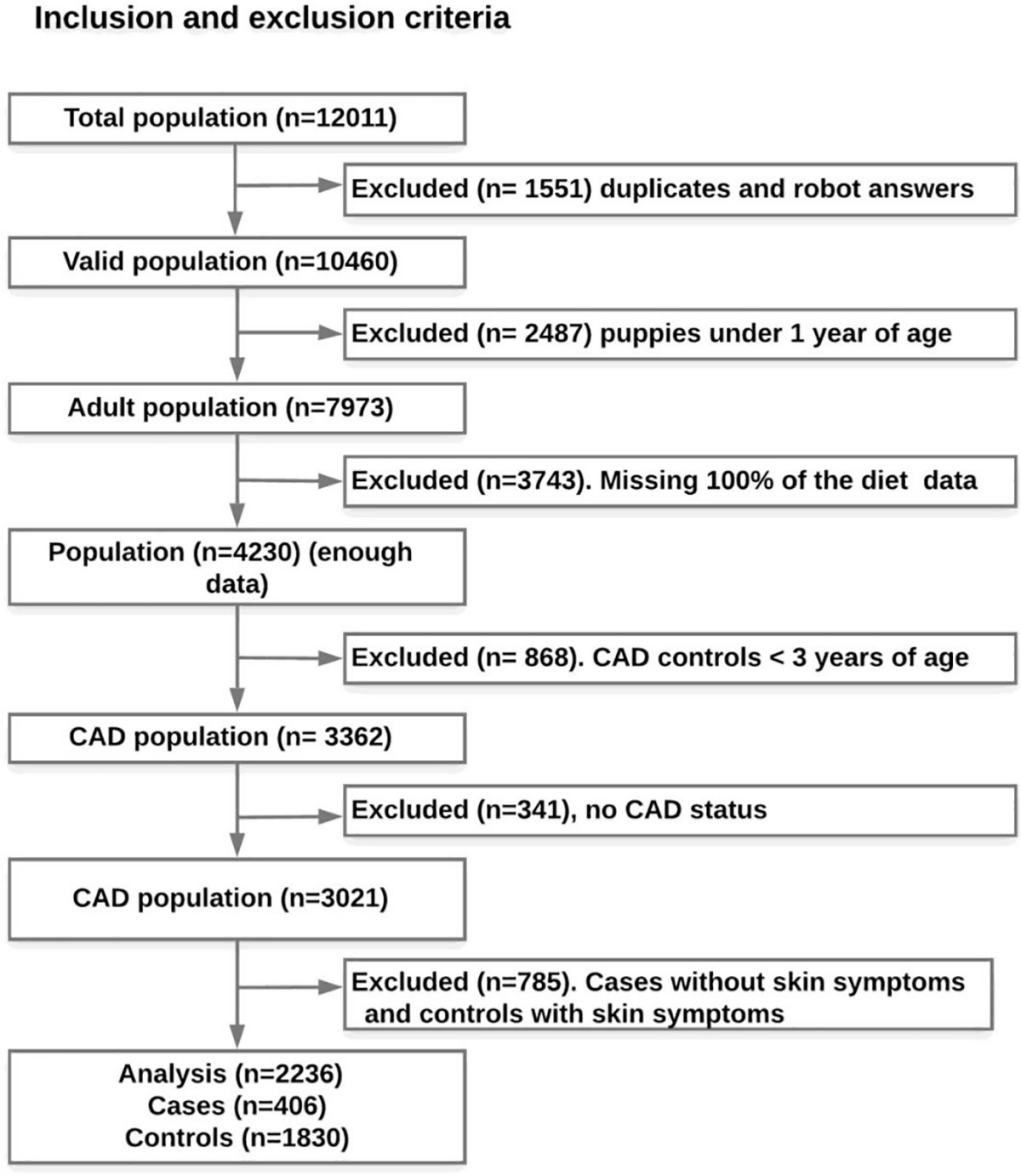
Flow chart of the study population. The study population was extracted from the DogRisk food frequency questionnaire population in the period between 2009 and 2018.

The total population consisted of 12,011 dogs (Fig 2). After excluding the duplicates and the robot answers (12.92%) our population consisted of 10,460 individuals. All breeds (allergy-prone and non-allergy prone) and both sexes (males and females) were eligible for this study. Then, according to study inclusion criteria, we handled the data as follows: We excluded all puppies under one year of age in order to avoid reverse causality [53], and further, excluded all dogs under 3 years of age from the control group to avoid cases that had not yet erupted, as the “age of disease onset” is considered to be 0–3 years of age [4]. Furthermore, the participants who did not respond to the questions about having CAD or not, and those who had not given any data about the early life diets, were excluded from this study (Fig 2). Finally, to avoid wrongly diagnosed dogs (as the diagnosis of CAD was based only on owner reported diagnosis) we only included CAD dogs that whose owners had also responded that their dogs had skin symptoms (n = 406) while the allowed controls had to report that they neither had CAD nor skin symptoms (n = 1830) giving us a final adult dog population for analysis of n = 2236. This study was carried out to test the association between CAD and different nutritional, environmental, genetic and demographic variables in the pre- and postnatal periods (Fig 1). Additionally, to pinpoint both potential risk and protective effect of a variable on CAD incidence we tested the association between each category in all variables with CAD.

### Selection of variables

Our main interest was on the modifiable dietary and environmental exposures within pre and postnatal life. The nutritional variables during the prenatal, neonatal and early postnatal life as well as the maternal deworming and vaccination in the prenatal life were testing the DOHaD hypothesis. The hygiene hypothesis was tested by using the nutritional variables in the early and postnatal life and the environmental exposures in the postnatal life. Also, the already known genetic and demographic factors were tested, including dog breed, gender, color and maternal history of CAD.

#### Newly created independent variables

A dichotomous variable for dog breeds was created including allergy-prone breeds and non- allergy-prone breeds. These breed lists were modified from a previous article [14] with data from CAD research [15, 16, 54–58] and are included as a supplementary S1 Table. Two questions (Q26-27) were about the maternal diet during the pregnancy and the lactation period and a third about the young puppy’s first solid diet (Q32), before two months of age. For this study we only used an answer to the question “Do you remember what (type of food had been used)?” where the owner had typed in the type of food used, themselves. From the owners’ answers, we selected only dogs that either had answered that they were using one of two extreme diets; either they used a raw or non-processed meat based diet (NPMD) or a dry or ultra-processed carbohydrate based diet (UPCD). NPMD and UPCD in our study also represent the two extreme groups of the most recently applicable NOVA classification system of human foods, the non-processed foods versus the ultra-processed foods [42, 43, 59]. However, the NPMD tested diets in our study are not totally identical to group 1 of the NOVA classification [59], as they might have been deep-frozen and might have contained supplements like salt, artificial vitamins, mineral premixes etc. The similarity is that they are not heat-treated (not warmed over 45° C).

The answers of the body condition score (Q31 and 36) at the age of 2 months and 6 months respectively, were as follows: very chubby, chubby, normal, slim and very slim. The first two categories were merged into one category of overweight dogs. The last two categories (slim and very slim dogs) were merged into a category of underweight dogs. As such, the variable contained three categories of overweight, normal, and underweight puppies. Later the underweight and overweight were combined, resulting in a dichotomous variable of normal or not normal.

The answer to how many hours the dog spent in sunlight (Q29 and 34) at the age of 2 months and 6 months, respectively, was in form of a continuous numeric variable. Based on preliminary cut point analyses the answer at two months was changed into the dichotomous variable of zero hours versus one hour or more (up to 24 h) of sunlight exposure per day. In the case of the older puppies (6 months of age), it was changed into the dichotomous variable of zero or one-hour sunlight exposure versus more than one-hour sunlight exposure per day.

In questions 30 and 35 we were asking for the kind of “floor” the puppy was living on at 2 and 6 months of age, respectively. The multiple choices were; mainly a slippery floor, mainly a non-slippery floor, outside slippery ice, dirt/lawn, newspaper, soft carpet / rugs, or I do not know. Due to our previous research on urban and rural environments [14] we divided these into a dichotomous variable of dirt/lawn floor and non-dirt/lawn floor.

All other questions and answers were used as such.

### Statistical analysis

The data was analyzed using IBM SPSS statistics for windows, version 25.0. Armonk, NY: IBM Corp. Package 'forest plot' [60] in R software version 3.5.1 [61] was used for visualizing odds ratios. The distribution of the variables within the cases and the controls was calculated using the crosstabs descriptive analyses. The prevalence of CAD cases was calculated using the participants who answered the question regarding CAD, including all breeds, all ages, and both sexes.

The variables were initially screened individually for a potential association with having CAD or not, using univariate logistic regression analysis. The variables with a p ≤ 0.2 were then included in the multiple logistic regression analysis. The multiple regression analyses were run in five models; one for the non-modifiable genetic and demographic variables and four models for the tested four early life periods modifiable variables, using the method enter. The first model included dog gender as a tested variable and the last four models were adjusted for the dog gender. Further, variable interactions were run and the interacted variables were assessed for associations with CAD using univariate logistic regression analysis. In the logistic regression analysis, univariate and multivariate, the model was run twice for the dichotomous variables or more than two times for the variables including more than two categories, using a different category as a reference each time.

The correlation between the variables in each model was tested using bivariate Pearson correlation to avoid multicollinearity. The correlations within the variables in each model were weak (≤ 0.1), except the correlation between the frequency of outdoor activity and hours of sunlight exposure in the early postnatal period that was ≥ 0.5. As such, the variable of outdoor activity was excluded from model 4. The missing values in our data have not been imputed and are handled by the listwise deletion in the program. Significance was considered when the p-value ≤ 0.05. The fitness of the logistic regression analysis was checked using the Omnibus test (a p-value < 0.05 being good), the Hosmer and Lemeshow test (> 0.05 being good) and the Nagelkerke´s R (where a larger value is better) [62, 63].

## Results

### Variable characteristics

The prevalence of atopic cases within the participants in the food frequency questionnaire by answering the question regarding CAD was 18.8%. The mean age ± SD of the total study population was 5.40 ± 2.84, while for the cases and controls the mean ages were 4.88 ± 2.71 and 5.70 ± 2.75, respectively. Variable characteristics and their distribution within the canine atopic dermatitis (CAD) cases and the non-atopic control dogs are shown in Table 1.

### Associations between the early life exposures and the incidence of CAD

**Fig 3:**
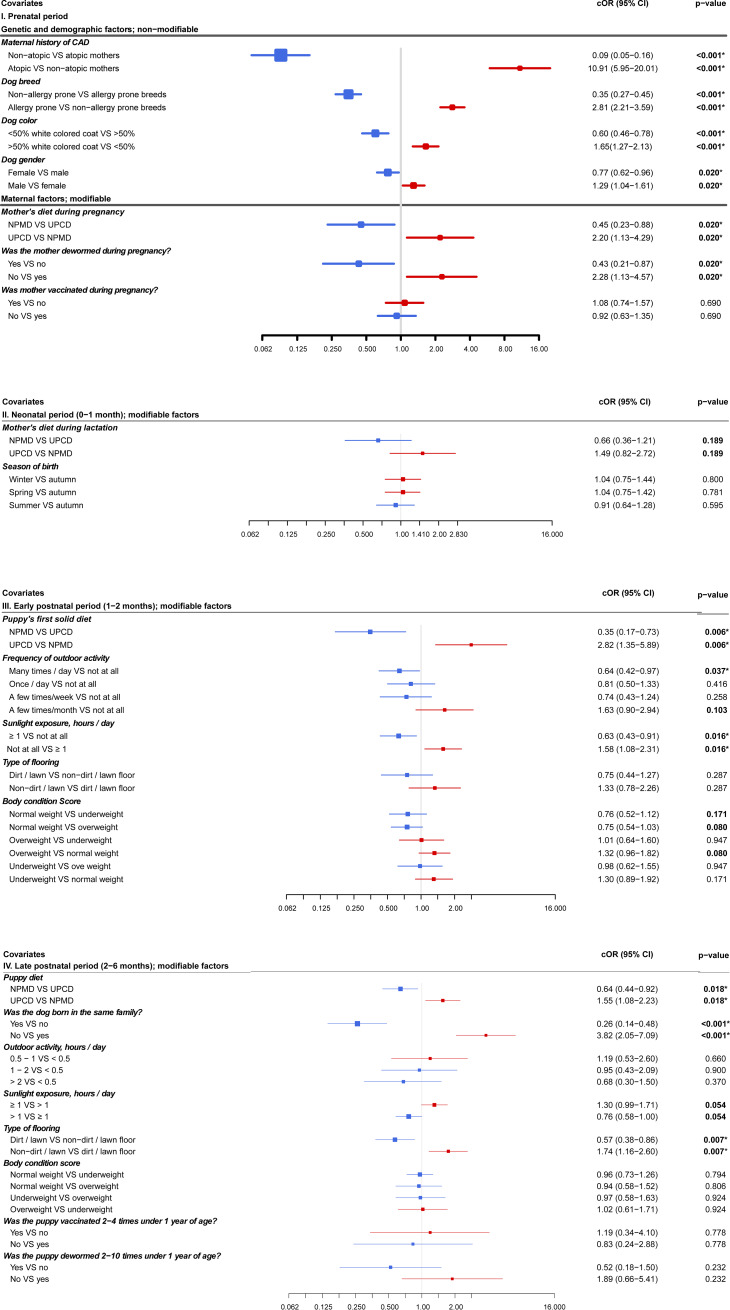
Forest plot of odds ratios for associations between pre- (A), neo- (B), early post- (C) and late postnatal (D) period variables and canine atopic dermatitis based on univariate logistic regression analyses (n = 2236). cOR: crude odds ratio, CI: confidence interval, CAD: canine atopic dermatitis, bolded: P ≤ 0.2, *: P ≤ 0.05, NPMD: non-processed meat based diet, UPCD: ultra-processed carbohydrate based diet, VS: versus.

From the univariate logistic regression analysis, we found that there were 12 variables from a total of 22 variables in the different periods of life, that were significantly associated (p ≤ 0.05) with CAD in dogs over one year of age. In Fig 3 and in the supplemental S2 Table we present odds ratios with confidence intervals OR (CI) for each category in all the variables. Blue and red lines represent decreased and increased risk when OR is below or above one, respectively (Fig 3). Contributing to open access, this also makes it easier for laymen potentially interested, to understand our results.

Univariate logistic regression analysis was done to assess the associations between the interacted variables and CAD incidence. No new significant associations were found between the interacted variables and CAD in comparison to the single variables and CAD.

**Fig 4:**
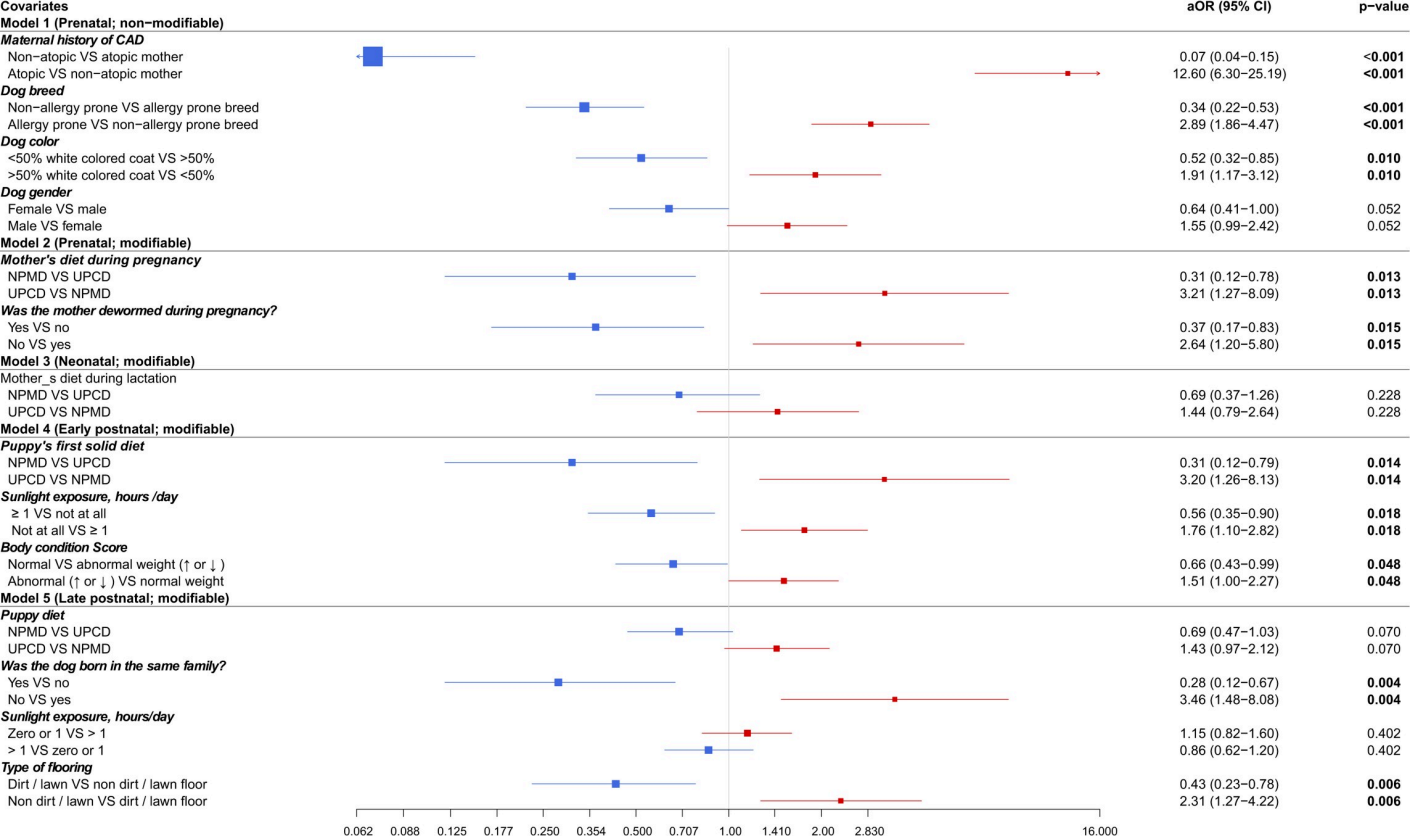
Forest plot of odds ratios for associations between pre-, neo-, early post- and late postnatal period variables and canine atopic dermatitis based on multivariable logistic regression analyses (n = 2236). Included/missing dogs for each model: model 1 (789/1447), model 2 (1357/879), model 3 (1297/939), model 4 (782/1454) and model 5 (1071/1165). Models 2–4 have been adjusted for dog gender. aOR: adjusted odds ratio, CI: confidence interval, CAD: canine atopic dermatitis, bolded: P ≤ 0.05, NPMD: non-processed meat based diet, UPCD: ultra-processed carbohydrate based diet, VS: versus.

The final models were created using multivariable logistic regression analysis. There were three significant variables in model 1 for the genetic and demographic non-modifiable variables, two significant variables in model 2 for the prenatal modifiable variables and three significant variables in model 4 for the early postnatal modifiable variables. In model 5 for the late postnatal modifiable variables there were two significant variables. Model 3 gave no significant factors. OR (CI) are given for each category in the dichotomous variables used in the five models as shown in Fig 4 and in the supplemental S3 Table. Blue and red colors in Fig 4 represent the decreased and increased risk when OR is below or above one, respectively.

## Discussion

In the current hypothesis generating study some modifiable early risk factors, nutritional and environmental, for CAD have been identified. Also, to verify the validity of our data, some genetic and background-related variables were also analyzed. We will discuss our findings against previous research and the data we have at hand.

### Genetic and background-related non-modifiable variables

The dogs with a maternal history of CAD were at high risk to develop CAD when becoming adult while those without a maternal history of CAD had a low chance of developing CAD during adulthood. However, as the offspring live in the same physical environment during their first 2 months and consume the mother’s milk, they are also usually first fed the same type of food (NPMD or UPCD) that the mother eats. The challenge hence remains to determine whether a multitude of genes are indeed the causative factor or whether it is the environment or the diet that matters more, possibly through altering relevant gene expression. In a previous study investigating the genetic predisposition of CAD in Labrador and Golden retrievers, they found that breeding two atopic parents resulted in 65% atopic offspring, breeding one atopic parent resulted in 21–57% atopic offspring and breeding two non- atopic parents resulted in 11% atopic offspring [12]. Unfortunately, there was no information provided regarding the environment or the diet of neither dams nor offspring. A recent human study showed that a family history of allergic disease is a risk factor for AD [64]. Also, Eichenfield et al. [65] reported that 70% of people with AD have a family history of atopy. Moreover, in our study the allergy prone breeds were associated with a high risk for CAD while the non-allergy prone breeds were protected from CAD. These findings are in accordance with Hakanen at al. [14], Anturaniemi et al. [13], Gedon and Mueller [66], and Jaeger et al. [55]. The dogs with a less than 50% white-colored coat were less susceptible to develop CAD while having a more than 50% white-colored coat put the dogs at high risk to develop CAD. This findings was in line with Anturaniemi et al. [13] and Nodtvedt et al. [41]. Almqvist et al. [17] found that gender is a contributing factor as boys are more susceptible to atopic sensitization than girls, although the mechanism is not clear. In our study we saw no difference in the final model which was in agreement with Anturaniemi [13].

### Prenatal modifiable variables

In the present study, having been subjected to NPMD during pregnancy resulted in a lower risk of CAD, while subjection to UPCD was associated with a higher risk of CAD in the offspring at adulthood. The potentially protective effects and risks of NPMD and UPCD can be attributed to many reasons. The dietary effect could be related to the time periods when they were served, their potentially healthy microbial load, the processing methods, and/or the macro- or micronutrient content.

To the extent of our knowledge, the study at hand is the first study to test the DOHaD hypothesis [27] on dogs. This “Developmental Origins of Health and Disease” concept, which is used extensively in observational human research [23, 33, 39, 67–74] analyzes the role of intrauterine and early postnatal nutrition on future health outcomes. Our results were consistent with the DOHaD hypothesis. The maternal diet during pregnancy is the only source of nutrition for the fetus as the fetus receives nutrients from its mother via the placenta. Any disturbance or imbalance of the maternal diet during gestation reflects on the newborn. This may result in an impaired immune system and high susceptibility to chronic diseases in the future [26].

As far as we know, the canine maternal gestation diet has not been analyzed before in conjunction with CAD. In a human study, Niinivirta-Joutsa [75] found interesting associations between maternal diet and the risk of allergy in children. He found a lower risk of atopic eczema in children whose mothers consumed milk and hen’s egg during pregnancy while consumption of cheese increased the risk. Moreover, antenatal exposure to maternal allergens was found to have an influence on the incidence of allergic diseases in genetically predisposed individuals [76]. But, in a conflicting study, there were no consistent associations between the maternal diet and the occurrence of atopy in their children, although some separate protective foods were reported [77].

The effect of the diet on allergy development depend on the kind of the diet, some diets are protective while others are a risk of allergy development [78]. Heat processing of UPCD has been shown to destroy nutrients, active enzymes and antioxidants, making it non-nutritionally sound [79]. Indeed, heat processing boosts the denaturation of food proteins, which interact with other food components, promoting immunogenicity and allergenicity [44]. On the other hand, the NPMD is highly palatable, the proteins and fats are highly digestible, it reduces blood triglycerides, maintains fecal quality and serum chemistry, and modifies the fecal microbiota community in adult dogs [80]. Moreover, eating a NPMD provides a variety of microbes when compared to eating UPCD, as the latter one is heat processed, which consequently kills the bacteria, making it sterile [40]. There is an indication that the transmission of microbiota from the intrauterine environment to the fetus may occur during pregnancy which seems to have a better impact on offspring future health [81].

Dogs are carnivores and have evolved on eating diets rich in animal proteins and fats (meats, fish, organs, edible bones etc.) as well as animal fiber (e.g. feather, scales, fur etc.) and low in carbohydrates [46, 47]. The UPCD contains high amounts of highly processed, and therefore highly fermentable, cereal grains (wheat, rice, oats, barley, rye, etc.) which are not a part of the canine ancestral diet. Cereal grains often contain gluten or other possibly harmful proteins [82]. Gluten intolerance or celiac disease occurs in humans and therefore gluten-containing grains are avoided by these patients [83, 84]. There are, however, very few studies or data on these diseases in dogs [85–92]. Gluten-related disorders might mimic the skin findings in humans and gluten avoidance seems to play a role in the prevention of AD [93]. As there are no minimum requirements for carbohydrates for dogs [94], a more species-appropriate, low or non-carbohydrate diet has been suggested to be used in cases of chronic skin diseases [95]. In a human study, the intake of allergenic foods such as margarine and vegetable oils during pregnancy increased the risk of allergic diseases in children, while consumption of foods rich in fish oil, decreased the risk [96]. However, discordant findings have been found when testing the effect of avoiding maternal dietary allergens in children with AD [97–101]. Furthermore, the Mediterranean diet during pregnancy seemed to be protective against allergic diseases in children [102, 103] which are in accordance with our results: the human Mediterranean diet contains more raw protein elements such as cured meats, raw shellfish, as well as raw eggs and raw vegetables, making them much more comparable to the NPMD of our study.

Other maternal factor tested in our study, maternal deworming during pregnancy, was negatively associated with CAD incidence in the puppies. This may indicate that deworming is essential to avoid the direct effects of worms on gastrointestinal pathology [104]. Moreover, helminth infection of the mother during pregnancy have been reported to have a long-lasting impact on the fetal immune system development and both risks and opportunities for diseases later [105]. The mother´s vaccination during pregnancy was non-significant in our study. In a previous study on dogs [13] neither of the variables were significant. In a human study [106], maternal inflammatory cytokines during pregnancy were associated with corresponding cytokines in children at one year of age, but did not associate with increased IgE or atopic dermatitis in children.

### Neonatal modifiable variables

The NPMD and UPCD failed to show any associations with CAD incidence in the offspring during the neonatal, i.e. lactation period. Thereby our negative results of an association between the mother’s lactation diet and adult CAD disagree with Nodtvedt et al. [41]. In another conflicting study, feeding the mother a non-commercial animal products during lactation was protecting its puppies from CAD, regardless of whether the puppy was eating commercial or non-commercial meat before 6 months of age [40]. The findings of maternal lactation diets and their association with allergic diseases are also conflicting in human epidemiological studies [107, 108]. In a meta-analysis of 27 prospective cohort human studies, there was no strong AD protective effect of exclusive breastfeeding for a minimum of 3 months, even among children with a positive AD family history [109]. Moreover, in another study there was no significant association between duration of exclusive breast-feeding and development of sensitization in the first 6 years of life in children [110].

The environmental variable during the neonatal period, season of birth, had no association with CAD incidence at adulthood neither in the univariate nor in the multivariate logistic regression analysis. In a previous study, season of birth was reported to have an effect: dogs born in autumn had a higher risk for CAD [111]. Furthermore, born in winter has been associated with development of AD in children [112].

### Early postnatal modifiable variables

In this period of life, puppies that consumed NPMD had a lower risk of CAD, while those that consumed UPCD were associated with a higher risk of CAD at adulthood. Postnatal nutrition is essential for optimal physiological development of vital organs [113]. Our findings for the early postnatal diet are also in line with the DOHaD hypothesis [23, 27]. In the early postnatal period, the newborn gut is exposed to food allergens for the first time. The acquired dietary microbiota and their interactions also stimulate the immune tolerance development [114, 115]. This also supports the hygiene hypothesis which states that early life exposure to dietary microbes influences the immune system development against atopic dermatitis [116]. In a human study, child adherence to the Mediterranean diet was protective against allergic diseases [117, 118] while child adherence to the western diet was a risk factor for AD [118]. Moreover, some farming environment studies have shown that unprocessed farm milk consumption by the child is associated with a lower prevalence of allergic disease, without depending on other farm-related covariates [119, 120]. The decreased and increased risk of CAD with the NPMD and UPCD have been interpreted above with the maternal diet during pregnancy.

Sunlight exposure for one or more than one hours per day during the early postnatal period (1–2 months of age) was significantly associated with lower risk of CAD in dogs at one year of age. Daily outdoor sunlight exposure in early life increases the exposure to both sun and external allergens. Some human research suggests that insufficient sunlight exposure in the first two years of life might increase the risks of development of AD [112] while exposure to ultraviolet sun light showed a positive significant association with AD symptoms in children [121]. In addition, the exposure to the outdoor environmental microbes has been found to influence the immune system against sensitization [14, 122]. The environmental exposures in the early life period are very critical. The hygiene hypothesis clarified that early exposure to microbiota is important in stimulating the immune tolerance in humans and this is probably also true for dogs. Recent studies in dogs showed an increased risk for CAD with urban environment and regular washing of healthy puppies, and a decreased risk of CAD with rural environment and forest walks [14, 122, 123].

The normal body weight of the young puppy before two months of age seems to be associated with lower odds of suffering from CAD while an increased or decreased body weight were both associated with a higher risk of CAD incidence in adulthood. In the Anturaniemi et al. [13] study the dogs that were obese or very slim at the age of 2 months were more susceptible to develop CAD when compared to the dogs with a normal body condition score. Recently, a positive association between a high body condition score/overweight and atopic dermatitis in cats [124] and in infants [125] have been reported. Moreover, our results also agreed with Zhang and Silverberg [126] who found an association between obesity and prevalence of AD in North America and Asia. Furthermore, longtime obesity in early life is a risk factor for atopic dermatitis and weight loss is essential for the prevention of atopic dermatitis in children [127]. This can be due to the low immunological tolerance in obese puppies [128].

### Late postnatal modifiable variables

The puppy diet consumed from 2 to 6 months of age associated with CAD but did not reach statistical significance. Similar to the other time frames the NPMD had a tendency to decrease the risk of CAD incidence in adulthood, while the UPCD tended to increase the risk. In a human study, high fish intake in late infancy was associated with a reduced AD risk [129, 130]. In contrast, Sallander et al.’s (2009) canine study found that feeding the puppy non-commercial meat before 6 months of age, had no effect on the risk of CAD later [40] but they only had 106 dogs compared to our over 2000 dogs. Also, the processing of the food was not clearly addressed and they just called the foods home-made diets / table diet. It is therefore possible that feeding only a meat-based diet might not be enough, it is possible that it also needs to be raw? So, for more evidence future prospective interventions are needed.

During 2–6 months of age, being born in the same family as it would proceed to continue living in, was associated with a lower risk of CAD in adulthood. These findings were also in accordance with our previous findings [13]. When the dog was born within the same family it meant that the dog continued to live in the same family where it was born and therefore it also continued to be exposed to at least one other dog, its mother. The protective effect of living with other dogs was in agreement with Meury et al. [122] and Fall et al. [131]. The protective effect is also in line with a human hygienic hypothesis study which emphasis that the more siblings, the less atopic dermatitis [132]. Moreover, when a dog continue to live in the same environment as it did in puppyhood, it would imply that the puppy already created immunity towards the external factors of that environment [13]. Another significant environmental factor during this period was the kind of flooring used by the puppy. We found a negative association between a dirt floor and CAD incidence in comparison with a non-dirt floor, which associated positively with CAD incidence. This was in accordance with Lehtimäki et al. [123] who found that exposure to arable land and forest in the surroundings of the birthplace associated with less allergic symptoms in dogs. The same has also been reported by Ruokolainen et al. [133] who found that early-life exposure to green environments (forest and agriculture land) is associated with lower atopic sensitization in 6 year old children. The environmental effect may be intermediated by the effect of land or floor microbiota on the commensal microbiota influencing immune tolerance.

## Strengths and limitations of the study

The strengths of this study are the use of validated data [52], using a wide range of potential covariates (nutritional, environmental and genetic), investigating four early life periods, and a satisfactory sample size (2236 dogs included in the analysis). Moreover, the reverse causality effect has been avoided here by excluding the puppies under the age of one year from the cases and under 3 years from the controls. Hereby we eliminate most patients that either eat something because of already having the disease and we eliminate controls that later might have turned into cases. Furthermore, this study is the first study to investigate the feeding pattern during the gestation period and its association with CAD incidence in the offspring, hence testing the DOHaD hypothesis.

However, our study, like any observational epidemiologic study, has some limitations. The first limitation is the study design. This is a cross-sectional study where the questions have been asked regarding different time points. This enabled us to use longitudinal data, but possibly with a bit less certitude, as memory comes into play. A second limitation is that the cases and controls were based on owners’ reports and not verified by professional veterinary diagnosing. This limitation, however, was amended by validating the questionnaire for CAD [52].

## Conclusion and recommendation

In conclusion, there were many modifiable variables that were associated with the prevalence of CAD: The NPMD during the prenatal and early postnatal periods suggested a protective effect on CAD, while the UPCD during the same periods suggested an increase of the risk of adult CAD incidence. In addition, deworming the mother dog during pregnancy, getting one hour or more of sunlight exposure per day at 1–2 months of age, having a normal body condition score at the age of 2 months, being born in the same family that the dog still lives in, and having spent time on a dirt/lawn floor at the age 2–6 months, were all associated with a significantly lower risk of CAD incidence in the adult dog. As predicted, the non-modifiable risk factors such as dogs with maternal history of CAD, from an allergy prone breed and with more than 50% white colored coat, were highly correlated with CAD. The modifiable risk factors, nutritional and environmental, during prenatal and postnatal periods provided by this study are critical starting points for further research. Prospective, randomized, longitudinal dietary intervention studies should now be undertaken both in dogs with a genetic predisposition, as well as in dogs without the genetic burden, to develop individualized primary prevention strategies for future prevention of CAD.

## Supporting information

S1 Table Breeds’ predisposition to develop canine atopic dermatitis. (DOCX)

S2 Table Associations between pre-, neo-, early post- and late postnatal period variables and canine atopic dermatitis based on univariate logistic regression analyses. (DOCX)

S3 Table Associations between pre-, neo-, early post- and late postnatal period variables and canine atopic dermatitis based on multivariate logistic regression analyses. (DOCX)

## Abbreviations

AD: atopic dermatitis (in humans
CAD: Canine atopic dermatitis
NPMD: non-processed meat based diet
UPCD: ultra-processed carbohydrate based diet
DOHaD: developmental origins of health and disease
FFQ: Food Frequency Questionnaire

## References

[1] Olivry T. International Task Force of Canine Atopic Dermatitis. New diagnostic criteria for canine atopic dermatitis. Vet Dermatol. 2010; 21(1): 123–126. 10.1111/j.1365-3164.2009.00776.x 20187919

[2] Gram D, Milner R, Lobetti R, editors. Chronic disease management for small animals. Hoboken, NJ: Wiley Blackwell, 2018; 23–38.

[3] Ka D, Marignac G, Desquilbet L, Freyburger L, Hubert B, Garelik D, et al Association between passive smoking and atopic dermatitis in dogs. Food Chem Toxicol. 2014; 66: 329–333. 10.1016/j.fct.2014.01.015 24491262

[4] Griffin CE, DeBoer DJ. The ACVD task force on canine atopic dermatitis (XIV): clinical manifestations of canine atopic dermatitis. Vet Immunol Immunopathol. 2001; 81(3–4): 255–269. 10.1016/s0165-2427(01)00346-4 11553388

[5] Martins LL, Bento OP, Inácio FF. Veterinary allergy diagnosis: past, present and future perspectives. Allergo J. 2016; 25(8): 20–32. 10.1007/s15007-016-1241-4

[6] Jordaan HF, Todd G, Sinclair W, Green RJ. Aetiopathogenesis of atopic dermatitis. S Afr Med J. 2014; 104(10): 706–709. 10.7196/samj.8840 25538994

[7] Bizikova P, Pucheu-Haston CM, Eisenschenk MN, Marsella R, Nuttall T, Santoro D. Review: Role of genetics and the environment in the pathogenesis of canine atopic dermatitis. Vet Dermatol. 2015; 26(2): 95–e26. 10.1111/vde.12198 25703290

[8] Hendaus MA, Jomha FA, Ehlayel M. Allergic diseases among children: Nutritional prevention and intervention. Ther Clin Risk Manag. 2016; 12: 361–372. 10.2147/TCRM.S98100 27022267 PMC4788360

[9] Roque JB, O’Leary CA, Duffy DL, Kyaw-Tanner M, Gharahkhani P, Vogelnest L, et al Atopic dermatitis in West Highland white terriers is associated with a 1.3-Mb region on CFA 17. Immunogenetics. 2012; 64: 209–217. 10.1007/s00251-011-0577-x 21989516

[10] Owczarek-Lipska M, Lauber B, Molitor V, Meury S, Kierczak M, Tengvall K, et al Two loci on chromosome 5 are associated with serum IgE levels in Labrador retrievers. PLoS ONE 2012; 7: e3917621 10.1371/journal.pone.0039176 22720065 PMC3376118

[11] Tengvall K, Kierczak M, Bergvall K, Olsson M, Frankowiack M, Farias FH, et al Genome-wide analysis in German shepherd dogs reveals association of a locus on CFA 27 with atopic dermatitis. PLoS Genet. 2015;11 (12):e1005740 10.1371/journal.pgen.1005740 26657407 PMC4676723

[12] Shaw SC, Wood JL, Freeman J, Littlewood JD, Hannant D. Estimation of heritability of atopic dermatitis in Labrador and Golden Retrievers. Am J Vet Res. 2004; 65(7): 1014–1020. 10.2460/ajvr.2004.65.1014 15281664

[13] Anturaniemi J, Uusitalo L, Hielm-Björkman A. Environmental and phenotype-related risk factors for owner-reported allergic/atopic skin symptoms and for canine atopic dermatitis verified by veterinarian in a Finnish dog population. PLoS One. 2017; 12(6): e0178771 10.1371/journal.pone.0178771 28570617 PMC5453595

[14] Hakanen E, Lehtimäki J, Salmela E, Tiira K, Anturaniemi J, Hielm-Björkman A, et al Urban environment predisposes dogs and their owners to allergic symptoms. Sci Rep. 2018; 8(1): 1585 10.1038/s41598-018-19953-3 29371634 PMC5785484

[15] Picco F, Zini E, Nett C, Naegeli C, Bigler B, Rüfenacht S, et al A prospective study on canine atopic dermatitis and food-induced allergic dermatitis in Switzerland. Vet Dermatol. 2008; 19(3): 150–155. 10.1111/j.1365-3164.2008.00669.x 18477331

[16] Zur G, Ihrke PJ, White SD, Kass PH. Canine atopic dermatitis: a retrospective study of 266 cases examined at the University of California, Davis, 1992–1998. Part I. Clinical features and allergy testing results. Vet Dermatol. 2002; 13(2): 89–102. 10.1046/j.1365-3164.2002.00285.x 11972892

[17] Almqvist C, Worm M, Leynaert B. Impact of gender on asthma in childhood and adolescence: a GA2LEN review. Allergy. 2008; 63(1): 47–57. 10.1111/j.1398-9995.2007.01524.x 17822448

[18] Herrmann I, Einhorn L, Panakova L. Gender aspects in allergies of pets—A secondary publication and update. World Allergy Organ J. 2017; 10(1): 42 10.1186/s40413-017-0172-1 29308109 PMC5746012

[19] Daigle J, Moussy A, Mansfield CD, Hermine O. Masitinib for the treatment of canine atopic dermatitis: A pilot study. Vet Res Commun. 2010; 34: 51–63. 10.1007/s11259-009-9332-2 20033487

[20] Cadot P, Hensel P, Bensignor E, Hadjaje C, Marignac G, Beco L, et al Masitinib decreases signs of canine atopic dermatitis: A multicentre, randomized, double-blind, placebo-controlled phase 3 trial. Vet Dermatol. 2011; 22: 554–564. 10.1111/j.1365-3164.2011.00990.x 21668810

[21] Leonard BC, Marks SL, Outerbridge CA, Affolter VK, Kananurak A, Young A, et al Activity, expression and genetic variation of canine beta-defensin 103: A multifunctional antimicrobial peptide in the skin of domestic dogs. J Innate Immun. 2012; 4: 248–259. 10.1159/000334566 22261569 PMC3357142

[22] van Damme CMM, Willemse T, van Dijk A, Haagsman HP, Veldhuizen EJA. Altered cutaneous expression of β-defensins in dogs with atopic dermatitis. Mol Immunol. 2009; 46: 2449–2455. 10.1016/j.molimm.2009.05.028 19576634

[23] Prescott SL. Early origins of allergic disease: a review of processes and influences during early immune development. Curr Opin Allergy Clin Immunol. 2003; 3(2): 125–132. 10.1097/00130832-200304000-00006 12750609

[24] Hanson M, Gluckman P. Developmental origins of noncommunicable disease: population and public health implications. Am J Clin Nutr. 2011; 94(6): 1754S–1758S. 10.3945/ajcn.110.001206 21525196

[25] Gluckman PD, Hanson MA, Cooper C, Thornburg KL. Effect of in utero and early life conditions on adult health and disease. N Engl J Med. 2008; 359(1): 61–73. 10.1056/NEJMra0708473 18596274 PMC3923653

[26] Barker DJP. The fetal and infant origins of adult disease. BMJ. 1990; 301(6761): 1111 10.1136/bmj.301.6761.1111 2252919 PMC1664286

[27] Gluckman P, Hanson M. Developmental origins of health and disease. Cambridge: Cambridge University Press; 2006 10.1017/CBO9780511544699

[28] Guilloteau P, Zabielski R, Hammon HM, Metges CC. Adverse effects of nutritional programming during prenatal and early postnatal life, some aspects of regulation and potential prevention and treatments. J Physiol Pharmacol. 2009; 60(3): 17–35. 19996479

[29] Mandy M, Nyirenda M. Developmental Origins of Health and Disease: the relevance to developing nations. Int Health 2018; 10: 66–70. 10.1093/inthealth/ihy006 29528398 PMC5856182

[30] Palmer DJ, Metcalfe J, Prescott SL. Preventing disease in the 21st century: The importance of maternal and early infant diet and nutrition. J Allergy Clin Immunol. 2012; 130(3): 733–734. 10.1016/j.jaci.2012.06.038 22857793

[31] Zhou X, Du L, Shi R, Chen Z, Zhou Y, Li Z. Early-life food nutrition, microbiota maturation and immune development shape life-long health. Crit Rev Food Sci Nutr. 2019; 59(1): S30–S38. 10.1080/10408398.2018.1485628 29874476

[32] Pai UA, Chandrasekhar P, Carvalho RS, Kumar S. The role of nutrition in immunity in infants and toddlers: An expert panel opinion. Clin Epidemiol Global Health. 2018; 6(4): 155–159. 10.1016/j.cegh.2017.11.004

[33] MacGillivray DM, Kollmann TR. The role of environmental factors in modulating immune responses in early life. Front Immunol. 2014; 5: 434 10.3389/fimmu.2014.00434 25309535 PMC4161944

[34] Martikainen MV, Rönkkö TJ, Schaub B, Täubel M, Gu C, Wong GW, et al Integrating farm and air pollution studies in search for immunoregulatory mechanisms operating in protective and high‐risk environments. Pediatr Allergy Immunol. 2018; 29(8): 815–822. 10.1111/pai.12975 30152886

[35] Lucas A. Programming by early nutrition in man. In: Bock GR, Chichester WJ, editors. The Childhood Environment and Adult Disease. Ciba Found Symp United States: Wiley 1991: 38–55. 1855415

[36] Heindel JJ. The developmental basis of disease: Update on environmental exposures and animal models. Basic Clin Pharmacol Toxicol. 2019; 125(3): 5–13. 10.1111/bcpt.13118 30265444

[37] Hattersley AT, Beards F, Ballantyne E, Appleton M, Harvey R, Ellard S. Mutations in the glucokinase gene of the fetus result in reduced birth weight. Nat Genet. 1998; 19(3): 268–270. 10.1038/953 9662401

[38] Amarasekera M, Prescott SL, Palmer DJ. Nutrition in early life, immune-programming and allergies: the role of epigenetics. Asian Pac J Allergy Immunol. 2013; 31(3): 175–182. 24053699

[39] Drake AJ, Walker BR. The intergenerational effects of fetal programming: non-genomic mechanisms for the inheritance of low birth weight and cardiovascular risk. J Endocrinol. 2004; 180(1): 1–16. 10.1677/joe.0.1800001 14709139

[40] Sallander M, Adolfsson J, Bergvall K, Hedhammar Å, Nodtvedt A. The effect of early diet on canine atopic dermatitis (CAD) in three high-risk breeds. The open Dermatol Journal. 2009; 3: 73–80. 10.2174/1874372200903010073

[41] Nødtvedt A, Bergvall K, Sallander M, Egenvall A, Emanuelson U, Hedhammar A. A case-control study of risk factors for canine atopic dermatitis among boxer, bullterrier and West Highland white terrier dogs in Sweden. Vet Dermatol. 2007; 18(5): 309–315. 10.1111/j.1365-3164.2007.00617.x 17845618

[42] Monteiro CA, Cannon G, Moubarac JC, Levy RB, Louzada MLC, Jaime PC. The UN Decade of Nutrition, the NOVA food classification and the trouble with ultra-processing. Public Health Nutr. 2018; 21(1): 5–17. 10.1017/S1368980017000234 28322183 PMC10261019

[43] Rico-Campà A, Martínez-González MA, Alvarez-Alvarez I, Mendonça RD, de la Fuente-Arrillaga C, Gómez-Donoso C, Bes-Rastrollo M. Association between consumption of ultra-processed foods and all-cause mortality: SUN prospective cohort study. BMJ. 2019; 365: l1949 10.1136/bmj.l1949 31142450 PMC6538973

[44] Teodorowicz M, van Neerven J, Savelkoul H. Food Processing: The Influence of the Maillard Reaction on Immunogenicity and Allergenicity of Food Proteins. Nutr. 2017; 9(8): 835 10.3390/nu9080835 . 28777346 PMC5579628

[45] Van Rooijen C, Bosch G, van der Poel AF, Wierenga PA, Alexander L, Hendriks WH. The Maillard reaction and pet food processing: effects on nutritive value and pet health. Nutr Res Rev. 2013; 26(2): 130–148. 10.1017/S0954422413000103 23916186

[46] Landry SM, Van Ruining HJ. The diet of feral carnivores: a review of stomach content analysis. J Am Anim Hosp Assoc. 1979; 15: 775–781.

[47] Coppinger R, Coppinger L. Dogs. In: A startling New Understanding of Canine Origin, Behavior and Evolution, Prentice, Hall and IBD: Scribner, New York 2001.

[48] Puotinen CJ. What a wolf eats: research on wild canids can help inform dietary planning for dogs. WDJ. 2005; 8(3).

[49] Brown S. The canine ancestral diet. In: Unlocking the Canine Ancestral Diet: Healthier Dog Food the ABC Way, Dogwise Publishing: Wenatchee, Washington 2010: 5–11.

[50] Woon FC, Chin YS, Ismail IH, Chan YM, Batterham M, Abdul Latiff AH, et al Contribution of early nutrition on the development of malnutrition and allergic diseases in the first year of life: a study protocol for the Mother and Infant Cohort Study (MICOS). BMC Pediatr. 2018; 18(1): 233 10.1186/s12887-018-1219-3 30021541 PMC6052551

[51] Strachan DP. Family size, infection and atopy: the first decade of the “hygiene hypothesis”. Thorax. 2000; 55(1): S2–10. 10.1136/thorax.55.suppl_1.s2 10943631 PMC1765943

[52] Roine J, Uusitalo L, Hielm-Bjorkman A. Validating and reliability testing the descriptive data and three different disease diagnoses of the internet-based DOGRISK questionnaire. BMC Vet Res. 2016; 12: 30 10.1186/s12917-016-0658-z 26897627 PMC4761135

[53] Roduit C. Development of atopic dermatitis and its association with prenatal and early life exposures. Doctoral thesis, Basel University, Faculty of Science. 2015. Available from: http://edoc.unibas.ch/diss/DissB_11324

[54] Bellumori TP, Famula TR, Bannasch DL, Belanger JM, Oberbauer AM. Prevalence of inherited disorders among mixed-breed and purebred dogs: 27,254 cases (1995–2010). J. Am. Vet. Med. Assoc. 2013; 242: 1549–1555. 23683021 10.2460/javma.242.11.1549

[55] Jaeger K, Linek M, Power HT, Bettenay SV, Zabel S, Rosychuk RA, et al Breed and site predispositions of dogs with atopic dermatitis: a comparison of five locations in three continents. Vet Dermatol. 2010; 21(1): 118–122. 10.1111/j.1365-3164.2009.00845.x 20187918

[56] Lund E. The epidemiology of atopic dermatitis. Banf. J. 2008; 4(2): 17–22.

[57] Nødtvedt A, Egenvall A, Bergval K, Hedhammar Å. Incidence of and risk factors for atopic dermatitis in a Swedish population of insured dogs. Vet Rec. 2006; 159(8): 241–246. 10.1136/vr.159.8.241 16921013

[58] Tarpataki N, Pápa K, Reiczigel J, Vajdovich P, Vörös K. Prevalence and features of canine atopic dermatitis in Hungary. Acta Vet. Hung. 2006; 54: 353–366. 17020139 10.1556/AVet.54.2006.3.6

[59] Monteiro CA, Cannon G, Lawrence M, Costa Louzada ML, Pereira Machado P. Ultra-processed foods, diet quality, and health using the NOVA classification system. Rome, FAO 2019.

[60] Gordon M, Lumley T. Advanced Forest Plot Using 'grid' Graphics. R package version 1.9, 2019. https://CRAN.R-project.org/package=forestplot

[61] R Core Team. R: A language and environment for statistical computing. R Foundation for Statistical Computing, Vienna, Austria 2017 https://www.R-project.org/

[62] Dohoo I, Martin W, Stryhn H, Hilbe J, Anthony J. Methods in epidemiologic research. Charlottetown, P.E.I: VER Inc. 2012: 413, 499–500.

[63] Hosmer DW, Lemeshow S. Applied logistic regression. 2nd ed New York: Wiley. Press 2000 10.1002/0471722146

[64] Acharya D, Bajgain BB, Yoo SJ. Factors Associated with Atopic Dermatitis and Allergic Rhinitis among Residents of Two Municipal Areas in South Korea. Medicina (Kaunas). 2019; 55(5): 13 10.3390/medicina55050131 31083640 PMC6572473

[65] Eichenfield LF, Tom WL, Chamlin SL, Feldman SR, Hanifin JM, Simpson EL, et al Guidelines of care for the management of atopic dermatitis: section 1. Diagnosis and assessment of atopic dermatitis. J Am Acad Dermatol. 2014; 70(2): 338–351. 10.1016/j.jaad.2013.10.010 24290431 PMC4410183

[66] Gedon NKY, Mueller RS. Atopic dermatitis in cats and dogs: a difficult disease for animals and owners. Clin Transl Allergy. 2018; 8: 41 10.1186/s13601-018-0228-5 30323921 PMC6172809

[67] Vickers MH. Early Life Nutrition, Epigenetics and Programming of Later Life Disease. Nutrients. 2014; 6(6): 2165–2178. 10.3390/nu6062165 24892374 PMC4073141

[68] Prescott SL. Early life environmental determinants of allergic diseases and the wider pandemic of inflammatory noncommunicable diseases. J Allergy Clin Immun. 2013; 131(1): 23–30. 10.1016/j.jaci.2012.11.019 23265694

[69] West CE, Jenmalm MC, Prescott SL. The gut microbiota and its role in the development of allergic disease: a wider perspective. Clin Exp Allergy. 2015; 45(1): 43–53. 10.1111/cea.12332 24773202

[70] Wang XM. Early life programming and metabolic syndrome. World J Pediatr. 2013; 9(1): 5–8. 10.1007/s12519-013-0403-7 23389329

[71] Wood CL, Wood AM, Harker C, Embleton ND. Bone Mineral Density and Osteoporosis after Preterm Birth: The Role of Early Life Factors and Nutrition. Int J Endocrinology. 2013; 2013: 902513 10.1155/2013/902513 23662104 PMC3639624

[72] Moore SE. Early life nutritional programming of health and disease in The Gambia. J Dev Orig Health Dis. 2016; 7(2): 123–131. 10.1017/S2040174415007199 26503192 PMC4825101

[73] Zheng J, Xiao X, Zhang Q, Yu M. DNA methylation: The pivotal interaction between early-life nutrition and glucose metabolism in later life. Br J Nutr. 2014; 112(11): 1850–1857. 10.1017/S0007114514002827 25327140

[74] Gowland RL. Entangled lives: Implications of the developmental origins of health and disease hypothesis for bioarchaeology and the life course. Am J Phys Anthropol. 2015; 158(4): 530–540. 10.1002/ajpa.22820 26767348

[75] Niinivirta-Joutsa K. Pre- and postnatal nutrition target for allergy risk reduction. Doctoral thesis, University of Turku. 2014. Available from: https://urn.fi/URN:ISBN:978-951-29-5729-3

[76] Warner JA, Jones CA, Jones AC, Warner JO. Prenatal origins of allergic disease. J Allergy Clin Immunol. 2000; 105(2 Pt 2): S493–498. 10.1016/s0091-6749(00)90049-6 10669530

[77] Netting MJ, Middleton PF, Makrides M. Does maternal diet during pregnancy and lactation affect outcomes in offspring? A systematic review of food-based approaches. Nutrition. 2014; 30(11–12): 1225–1241. 10.1016/j.nut.2014.02.015 25280403

[78] Varraso R, Camargo CA. Diet and asthma: Need to account for asthma type and level of prevention. Expert Rev Respir Med. 2016; 10(11): 1147–1150. 10.1080/17476348.2016.1240033 27701925

[79] Satpute M, Annapure U. Approaches for delivery of heat sensitive nutrients through food systems for selection of appropriate processing techniques: a review. J Hyg Eng Design. 2013; 4: 71–92.

[80] Algya KM, Cross TL, Leuck KN, Kastner ME, Baba T, Lye L, et al Apparent total-tract macronutrient digestibility, serum chemistry, urinalysis, and fecal characteristics, metabolites and microbiota of adult dogs fed extruded, mildly cooked, and raw diets. J Anim Sci. 2018; 96(9): 3670–3683. 10.1093/jas/sky235 29893876 PMC6127788

[81] Chu DM, Meyer KM, Prince AL, Aagaard KM. Impact of maternal nutrition in pregnancy and lactation on offspring gut microbial composition and function. Gut Microbes. 2016; 7(6): 459–470. 10.1080/19490976.2016.1241357 27686144 PMC5103658

[82] Sapone A, Bai JC, Ciacci C, Dolinsek J, Green PH, Hadjivassiliou M, et al Spectrum of gluten-related disorders: consensus on new nomenclature and classification. BMC Med. 2012; 10: 13 10.1186/1741-7015-10-13 22313950 PMC3292448

[83] Rubio-Tapia A, Hill ID, Kelly CP, Calderwood AH, Murray JA. ACG clinical guidelines: diagnosis and management of celiac disease. Am J Gastroenterol. 2013; 108(5): 656–676; quiz 677. 10.1038/ajg.2013.79 23609613 PMC3706994

[84] Silvester JA, Graff LA, Rigaux L, Walker JR, Duerksen DR. Symptomatic suspected gluten exposure is common among patients with coeliac disease on a gluten-free diet. Aliment Pharmacol Ther. 2016; 44(6): 612–619. 10.1111/apt.13725 27443825 PMC5283559

[85] Hall EJ, Batt RM. Dietary modulation of gluten sensitivity in a naturally occurring enteropathy of Irish setter dogs. Gut. 1992; 33(2): 198–205. 10.1136/gut.33.2.198 1347279 PMC1373930

[86] Hall EJ, Batt RM. Delayed introduction of dietary cereal may modulate the development of gluten-sensitive enteropathy in Irish setter dogs. J Nutr. 1991; 121(11): S152–S153. 10.1093/jn/121.suppl_11.S152 1941212

[87] Hall EJ, Batt RM. Abnormal intestinal permeability could play a role in the development of gluten-sensitive enteropathy in Irish setter dogs. J Nutr. 1991; 121(11): S150–S151. 10.1093/jn/121.suppl_11.S150 ] 1941211

[88] Hall EJ, Batt RM. Differential sugar absorption for the assessment of canine intestinal permeability: the cellobiose/mannitol test in gluten-sensitive enteropathy of Irish setters. Res Vet Sci. 1991; 51(1): 83–87. 10.1016/0034-5288(91)90036-n 1910201

[89] Hall EJ, Batt RM. Abnormal permeability precedes the development of a gluten sensitive enteropathy in Irish setter dogs. Gut. 1991; 32(7): 749–753. 10.1136/gut.32.7.749 1906829 PMC1378989

[90] Batt RM, McLean L, Carter MW. Sequential morphologic and biochemical studies of naturally occurring wheat-sensitive enteropathy in Irish setter dogs. Dig Dis Sci. 1987; 32(2): 184–194. 10.1007/BF01297107 3026759

[91] Batt RM, Carter MW, McLean L. Wheat-sensitive enteropathy in Irish setter dogs: possible age-related brush border abnormalities. Res Vet Sci. 1985; 39(1): 80–83. 2863858

[92] Polvi A, Garden OA, Houlston RS, Maki M, Batt RM, Partanen J. Genetic susceptibility to gluten sensitive enteropathy in Irish setter dogs is not linked to the major histocompatibility complex. Tissue Antigens. 1998; 52(6): 543–549. 10.1111/j.1399-0039.1998.tb03085.x 9894853

[93] Sur M, Aldea A, Dascal L, Duca E, Silaghi C, Sur L, et al The Link between the Clinical Features of Atopic Dermatitis and Gluten-related Disorders. Int J Celiac Dis. International Journal of Celiac Disease. 2019; 7(1): 31–32. 10.12691/ijcd-7-1-6

[94] Gross K, Wedekind KJ, Cowell CS, Schoenherr WD, Jewell DE, Zicker SC, et al In: Small Animal Clinical Nutrition, 4th edition, eds: Hand MS, Thatcher CD, Remillard RL, Roudebush P. Walsworth Publishing Company: Mark Morris Institute, Topeka 2000; 2: 38.

[95] Craig JM. Atopic dermatitis and the intestinal microbiota in humans and dogs. Vet Med Sci. 2016; 2(2): 95–105. 10.1002/vms3.24 29067183 PMC5645856

[96] Sausenthaler S, Koletzko S, Schaaf B, Lehmann I, Borte M, Herbarth O, et al Maternal diet during pregnancy in relation to eczema and allergic sensitization in the offspring at 2 y of age. Am J Clin Nutr. 2007; 85(2): 530–537. 10.1093/ajcn/85.2.530 17284754

[97] Falth-Magnusson K, Kjellman NI. Allergy prevention by maternal elimination diet during late pregnancy-a 5-year follow-up of a randomized study. J Allergy Clin Immunol. 1992; 89(3): 709–713. 10.1016/0091-6749(92)90378-f 1545092

[98] Herrmann ME, Dannemann A, Gruters A, Radisch B, Dudenhausen JW, Bergmann R, et al Prospective study of the atopy preventive effect of maternal avoidance of milk and eggs during pregnancy and lactation. Eur J Pediatr. 1996; 155(9): 770–774. 10.1007/BF02002904 8874109

[99] Hourihane JO, Dean TP, Warner JO. Peanut allergy in relation to heredity, maternal diet, and other atopic diseases: results of a questionnaire survey, skin prick testing, and food challenges. BMJ 1996; 313(7056): 518–521. 10.1136/bmj.313.7056.518 8789975 PMC2351952

[100] Lilja G, Dannaeus A, Foucard T, Graff-Lonnevig V, Johansson SG, Oman H. Effects of maternal diet during late pregnancy and lactation on the development of IgE and egg- and milk-specific IgE and IgG antibodies in infants. Clin Exp Allergy. 1991; 21(2): 195–202. 10.1111/j.1365-2222.1991.tb00830.x 2043987

[101] Lovegrove JA, Hampton SM, Morgan JB. The immunological and long-term atopic outcome of infants born to women following a milk-free diet during late pregnancy and lactation: a pilot study. Br J Nutr. 1994; 71(2): 223–238. 10.1079/bjn19940129 8142334

[102] De Batlle J, Garcia-Aymerich J, Barraza-Villarreal A, Antó JM, Romieu I. Mediterranean diet is associated with reduced asthma and rhinitis in Mexican children. Allergy. 2008; 63(10): 1310–1316. 10.1111/j.1398-9995.2008.01722.x 18782109

[103] Chatzi L, Torrent M, Romieu I, Garcia-Esteban R, Ferrer C, Vioque J, et al Mediterranean diet in pregnancy is protective for wheeze and atopy in childhood. Нorax. 2008; 63(6): 507–513 10.1136/thx.2007.081745 18198206

[104] Ndibazza J, Muhangi L, Akishule D, et al Effects of deworming during pregnancy on maternal and perinatal outcomes in Entebbe, Uganda: a randomized controlled trial. Clin Infect Dis. 2010; 50(4): 531–540. 10.1086/649924 20067426 PMC2857962

[105] Elliott AM, Kizza M, Quigley MA, Ndibazza J, Nampijja M, Muhangi L, et al The impact of helminths on the response to immunization and on the incidence of infection and disease in childhood in Uganda: design of a randomized, double-blind, placebo-controlled, factorial trial of deworming interventions delivered in pregnancy and early childhood. Clin Trials. 2007; 4(1): 42–57. 10.1177/1740774506075248 17327245 PMC2643383

[106] Herberth G, Hinz D, Röder S, Schlink U, Sack U, Diez U, et al Maternal immune status in pregnancy is related to offspring’s immune responses and atopy risk. Allergy. 2011; 66(8): 1065–1074. 10.1111/j.1398-9995.2011.02587.x 21443636

[107] Matheson MC, Allen KJ, Tang ML. Understanding the evidence for and against the role of breastfeeding in allergy prevention. Clin Exp Allergy. 2012; 42(6): 827–851. 10.1111/j.1365-2222.2011.03925.x 22276526

[108] Flohr C, Nagel G, Weinmayr G, Kleiner A, Strachan DP, Williams HC. Lack of evidence effect of prolonged breastfeeding on childhood eczema: Lessons from the International Study of Asthma and Allergies in Childhood (ISAAC) Phase Two. Br J Dermatol. 2011; 165(6): 1280–1289. 10.1111/j.1365-2133.2011.10588.x 21883137

[109] Yang YW, Tsai CL, Lu CY. Exclusive breastfeeding and incident atopic dermatitis in childhood: a systematic review and meta‐analysis of prospective cohort studies. Br J Dermatol. 2009; 161(2): 373–383. 10.1111/j.1365-2133.2009.09049.x 19239469

[110] Jelding-Dannemand E, Malby SAM, Bisgaard H. Breast-feeding does not protect against allergic sensitization in early childhood and allergy-associated disease at age 7 years. J Allergy Clin Immunol. 2015; 136(5): 1302–1308. 10.1016/j.jaci.2015.02.023 25843315

[111] Nødtvedt A, Egenvall A, Bergval K, Hedhammar Å. Incidence of and risk factors for atopic dermatitis in a Swedish population of insured dogs. Vet Rec. 2006; 159(8): 241–246. 10.1136/vr.159.8.241 16921013

[112] Hwang JM, Oh SH, Shin MY. The relationships among birth season, sunlight exposure during infancy, and allergic disease. Korean J Pediatr. 2016; 59(5): 218–225. 10.3345/kjp.2016.59.5.218 27279886 PMC4897157

[113] Caron E, Ciofi P, Prevot V, Bouret SG. Alteration in neonatal nutrition causes perturbations in hypothalamic neural circuits controlling reproductive function. J Neurosci. 2012; 32(33): 11486–11494. 10.1523/JNEUROSCI.6074-11.2012 22895731 PMC3460805

[114] Frei R, Lauener RP, Crameri R, O’Mahony L. Microbiota and dietary interactions: an update to the hygiene hypothesis? Allergy. 2012; 67(4): 451–461. 10.1111/j.1398-9995.2011.02783.x 22257145

[115] De Filippo C, Cavalieri D, Di Paola M, Ramazzotti M, Poullet JB, Massart S, et al Impact of diet in shaping gut microbiota revealed by a comparative study in children from Europe and rural Africa. Proc Natl Acad Sci USA. 2010; 107(33): 14691–14696. 10.1073/pnas.1005963107 20679230 PMC2930426

[116] Flohr C, Pascoe D, Williams HC. Atopic dermatitis and the 'hygiene hypothesis': too clean to be true? Br J Dermatol. 2005; 152(2): 202–216. 10.1111/j.1365-2133.2004.06436.x 15727630

[117] Chatzi L, Apostolaki G, Bibakis I, Skypala I, Bibaki-Liakou V, Tzanakis N, et al Protective effect of fruits, vegetables and the Mediterranean diet on asthma and allergies among children in Crete. Нorax. 2007; 62(8): 677–683. 10.1136/thx.2006.069419 PMC2117278 17412780

[118] Odette L, Pascale S, Chantal R, Celina B, Sarah K, Danielle S. The Association between Diet and Symptoms of Allergic Diseases in Children Aged from 8 to 12 in Public Schools in Beirut and Mount Lebanon. Pediatr Ther. 2018; 8(3): 349 10.4172/2161-0665.1000349

[119] Waser M, Michels KB, Bieli C, Floistrup H, Pershagen G, Von Mutius E, et al Inverse association of farm milk consumption with asthma and allergy in rural and suburban populations across Europe. Clin Exp Allergy. 2007; 37(5): 661–670. 10.1111/j.1365-2222.2006.02640.x 17456213

[120] Perkin MR, Strachan DP. Which aspects of the farming lifestyle explain the inverse association with childhood allergy? J Allergy Clin Immunol. 2006; 117(6): 1374–1381. 10.1016/j.jaci.2006.03.008 16751000

[121] Ahn K, Kim J, Jeon H, Kim H, Han Y, Jung K, et al Ultraviolet Sun Exposure Is Associated with the Acute Symptoms of Atopic Dermatitis in Young Children. J Allergy Clin Immunol. 2016; 137(2): AB153 10.1016/j.jaci.2015.12.630

[122] Meury S, Molitor V, Doherr MG, Roosje P, Leeb T, Hobi S, et al Role of the environment in the development of canine atopic dermatitis in Labrador and golden retrievers. Vet Dermatol. 2011; 22(4): 327–334. 10.1111/j.1365-3164.2010.00950.x 21251098

[123] Lehtimäkia J, Sinkkoa H, Hielm-Björkman A, Salmelaa E, Tiira K, Laatikainend T, et al Skin microbiota and allergic symptoms associate with exposure to environmental microbes. Proc Natl Acad Sci USA. 2018; 115(19): 4897–4902. 10.1073/pnas.1719785115 29686089 PMC5948976

[124] Teng KT, McGreevy PD, Toribio JALML, Raubenheimer D, Kendall K, Dhand NK. Associations of body condition score with health conditions related to overweight and obesity in cats. J Small Anim Pract. 2018; 59: 603–615. 10.1111/jsap.12905 30033652

[125] Berents TL, Carlsen KCL, Mowinckel P, Skjerven HO, Rolfsjord LB, Nordhagen LS, et al Weight-for-length, early weight-gain velocity and atopic dermatitis in infancy and at two years of age: a cohort study. BMC Pediatr. 2017; 17(1): 141 10.1186/s12887-017-0889-6 28592289 PMC5463398

[126] Zhang A, Silverberg JI. Association of atopic dermatitis with being overweight and obese: a systematic review and metaanalysis. J Am Acad Dermatol. 2015; 72(4): 606–616. 10.1016/j.jaad.2014.12.013 25773409

[127] Silverberg JI, Kleiman E, Lev-Tov H, Silverberg NB, Durkin HG, Joks R, Smith-Norowitz TA. Association between obesity and atopic dermatitis in childhood: a case-control study. J Allergy Clin Immunol. 2011; 127(5): 1180–1186. 10.1016/j.jaci.2011.01.063 21411132

[128] Cortese L, Terrazzano G, Pelagalli A. Leptin and Immunological Profile in Obesity and Its Associated Diseases in Dogs. Int J Mol Sci. 2019; 20 (10): 2392 10.3390/ijms20102392 PMC6566566 31091785

[129] Alm B, Aberg N, Erdes L, Möllborg P, Pettersson R, Norvenius SG, et al Early introduction of fish increases the risk of eczema in infants. Arch Dis Child. 2009; 94(1): 11–15. 10.1136/adc.2008.140418 18818269 PMC2597687

[130] Oien T, Storro O, Johnsen R. Do early intake of fish and fish oil protect against eczema and doctor diagnosed asthma at 2 years of age? A cohort study. J Epidemiol Community Health. 2010; 64(2): 124–129. 10.1136/jech.2008.084921 19666630

[131] Fall T, Lundholm C, Örtqvist AK, Fall K, Fang F, Hedhammar Å, et al Early Exposure to Dogs and Farm Animals and the Risk of Childhood Asthma. JAMA Pediatr. 2015; 169(11): e153219 10.1001/jamapediatrics.2015.3219 26523822

[132] Strachan DP. Hay fever, hygiene, and household size. BMJ 1989; 299(6710): 1259–1260. 10.1136/bmj.299.6710.1259 2513902 PMC1838109

[133] Ruokolainen L, von Hertzen L, Fyhrquist N, Laatikainen T, Lehtomäki J, Auvinen P, et al Green areas around homes reduce atopic sensitization in children. Allergy. 2015; 70(2): 195–202. 10.1111/all.12545 25388016 PMC4303942

