# Supplementary material for: Identification of modifiable pre- and postnatal dietary and environmental exposures associated with owner-reported canine atopic dermatitis in Finland using a web-based questionnaire
Source: PLoS One. 2020 May 29;15(5):e0225675. doi: 10.1371/journal.pone.0225675 (PMC7259748; doi:10.1371/journal.pone.0225675)
Supplement: S1 Table — (DOCX) [file pone.0225675.s001.docx]

**S1 Table. Breeds’ predisposition to develop canine atopic dermatitis.**

| **Authors** | **Canine atopic dermatitis prone breeds** |
| --- | --- |
| ***Bellumori et al. 2013*** | West Highland White Terrier, Cairn Terrier, Tibetan Terrier, Fox Terrier Wire |
| ***Jaeger et al. 2010*** | West Highland White Terrier, Boxer, German Shepherd Dog, Bull Terrier, Labrador Retriever, Golden Retriever, Great Dane, Cavalier King Charles  Spaniel, Australian Silky Terrier, French bulldog, Jack Russel Terrier, Bichon Frisé |
| ***Lund 2008*** | West Highland White Terrier, Boxer, Staffordshire Bull Terrier, Jack Russel Terrier, Cairn Terrier, Bichon Frisé, American Bulldog, Bulldog, Boston Terrier, Havanese, Miniature Schnauzer, Schnauzer, Shih Tzu, Maltese, Yorkshire Terrier, Lhasa Apso |
| ***Picco et al. 2008*** | West Highland White Terrier, Boxer, Bull Terrier, French bulldog, Rhodesian Ridgeback, Vizsla |
| ***Nødtvedt et al. 2006*** | West Highland White Terrier, Boxer, German Shepherd Dog, Bull Terrier, Labrador Retriever, American Staffordshire Terrier, Dalmatian, Rhodesian Ridgeback, Staffordshire Bull Terrier, Newfoundland, Danish-Swedish Farmdog, Irish Soft Coated Wheaten Terrier, Welsh Springer Spaniel, Welsh Terrier |
| ***Tarpataki et al. 2006*** | West Highland White Terrier, Boxer, German Shepherd Dog, French bulldog, American Staffordshire Terrier, Dalmatian, Vizsla, Newfoundland, Puli, English Setter, Dobermann, Poodle, Chow Chow, Pumi, Old English Sheepdog, Dachshund, English Cocker Spaniel |
| ***Zur et al. 2002*** | Labrador Retriever, Golden Retriever, German Shepherd Dog, American Cocker Spaniel, West Highland White Terrier, English Springer Spaniel, Dachshund, Doberman Pinscher, Chinese Shar-Pei, Dalmatian, Bull Terrier, Bichon Frisé, Tibetan Terrier, Mixed-breed dogs |
