# Supplementary material for: Identification of modifiable pre- and postnatal dietary and environmental exposures associated with owner-reported canine atopic dermatitis in Finland using a web-based questionnaire
Source: PLoS One. 2020 May 29;15(5):e0225675. doi: 10.1371/journal.pone.0225675 (PMC7259748; doi:10.1371/journal.pone.0225675)
Supplement: S2 Table — (DOCX) [file pone.0225675.s002.docx]

**S2 Table. Associations between pre-, neo-, early post- and late postnatal period variables and canine atopic dermatitis based on univariate logistic regression analyses.**

| **Covariates** | **Total population for analysis (n=2236)** | | **Crude effect estimates** | |
| --- | --- | --- | --- | --- |
|  | **Included dogs (n)** | **Missing dogs (n)** | **cOR (95% CI)** | **P-value** |
| **I. Prenatal period** |  |  |  |  |
| **Genetic and demographic factors; non-modifiable** | | | | |
| ***Maternal history of CAD*** | 1042 | 1194 |  |  |
| Non-atopic VS atopic mother |  |  | 0.09 (0.05-0.16) | **<0.001*** |
| Atopic VS non-atopic mother |  |  | 10.91 (5.95-20.01) | **<0.001*** |
| ***Dog breed*** | 1796 | 440 |  |  |
| Non-allergy prone VS allergy prone  breed |  |  | 0.35 (0.27-0.45) | **<0.001*** |
| Allergy prone VS non-allergy prone  breeds |  |  | 2.81 (2.21-3.59) | **<0.001*** |
| ***Dog color*** | 2096 | 140 |  |  |
| <50% white colored coat VS >50% |  |  | 0.60 (0.46-0.78) | **<0.001*** |
| >50% white colored coat VS <50% |  |  | 1.65 (1.27-2.13) | **<0.001*** |
| ***Dog gender*** | 2177 | 59 |  |  |
| Female VS male |  |  | 0.77 (0.62-0.96) | **0.020*** |
| Male VS female |  |  | 1.29 (1.04-1.61) | **0.020*** |
| **Maternal factors; modifiable** | | | | |
| ***Mother’s diet during pregnancy*** | 1389 | 847 |  |  |
| NPMD VS UPCD |  |  | 0.45 (0.23-0.88) | **0.020*** |
| UPCD VS NPMD |  |  | 2.20 (1.13-4.29) | **0.020*** |
| ***Was the mother dewormed during pregnancy?*** | 1395 | 841 |  |  |
| Yes VS no |  |  | 0.43 (0.21-0.87) | **0.020*** |
| No VS yes |  |  | 2.28 (1.13-4.57) | **0.020*** |
| ***Was mother vaccinated during pregnancy?*** | 803 | 1433 |  |  |
| Yes VS no |  |  | 1.08 (0.74-1.57) | 0.690 |
| No VS yes |  |  | 0.92 (0.63-1.35) | 0.690 |
| **II. Neonatal period (0-1 month); modifiable factors** | | | | |
| ***Mother’s diet during lactation*** | 1330 | 906 |  |  |
| NPMD VS UPCD |  |  | 0.66 (0.36-1.21) | **0.189** |
| UPCD VS NPMD |  |  | 1.49 (0.82-2.72) | **0.189** |
| ***Season of birth*** | 2202 | 34 |  |  |
| Winter VS autumn |  |  | 1.043 (0.75-1.44) | 0.800 |
| Spring VS autumn |  |  | 1.045 (0.75-1.42) | 0.781 |
| Summer VS autumn |  |  | 0.91 (0.64-1.28) | 0.595 |
| **III. Early postnatal period (1-2 months); modifiable factors** | | | | |
| ***Puppy’s first solid diet*** | 1360 | 876 |  |  |
| NPMD VS UPCD |  |  | 0.35 (0.17-0.73) | **0.006*** |
| UPCD VS NPMD |  |  | 2.82 (1.35-5.89) | **0.006*** |
| ***Frequency of outdoor activity*** | 1713 | 523 |  |  |
| Many times / day VS not at all |  |  | 0.64 (0.42-0.97) | **0.037*** |
| Once/day VS not at all |  |  | 0.81 (0.50-1.33) | 0.416 |
| A few times / week VS not at all |  |  | 0.74 (0.43-1.24) | 0.258 |
| A few times / month VS not at all |  |  | 1.63 (0.90-2.94) | **0.103** |
| ***Sunlight exposure, hours / day*** | 1215 | 1021 |  |  |
| ≥ 1 VS not at all |  |  | 0.63 (0.43-0.91) | **0.016*** |
| Not at all VS ≥ 1 |  |  | 1.58 (1.08-2.31) | **0.016*** |
| ***Type of flooring*** | 1775 | 461 |  |  |
| Dirt / lawn VS non-dirt / lawn floor |  |  | 0.75 (0.44-1.27) | 0.287 |
| Non-dirt / lawn VS dirt / lawn floor |  |  | 1.33 (0.78-2.26) | 0.287 |
| ***Body condition Score*** | 1888 | 348 |  |  |
| Normal weight VS underweight |  |  | 0.76 (0.52-1.12) | **0.171** |
| Normal weight VS overweight |  |  | 0.75 (0.54-1.03) | **0.080** |
| Overweight VS underweight |  |  | 1.01 (0.64-1.60) | 0.947 |
| Overweight VS normal weight |  |  | 1.32 (0.96-1.82) | **0.080** |
| Underweight VS over weight |  |  | 0.98 (0.62-1.55) | 0.947 |
| Underweight VS normal weight |  |  | 1.30 (0.89-1.92) | **0.171** |
| **IV. Late postnatal period (2-6 months); modifiable factors** | | | | |
| ***Puppy diet*** | 1287 | 949 |  |  |
| NPMD VS UPCD |  |  | 0.64 (0.44-0.92) | **0.018*** |
| UPCD VS NPMD |  |  | 1.55 (1.08-2.23) | **0.018*** |
| ***Was the dog born in the same family?*** | 2236 | 0 |  |  |
| Yes VS no |  |  | 0.26 (0.14-0.48) | **<0.001*** |
| No VS yes |  |  | 3.82 (2.05-7.09) | **<0.001*** |
| ***Outdoor activity, hours / day*** | 1853 | 383 |  |  |
| 0.5 - 1 VS < 0.5 |  |  | 1.19 (0.53-2.60) | 0.660 |
| 1 - 2 VS < 0.5 |  |  | 0.95 (0.43-2.09) | 0.900 |
| > 2 VS < 0.5 |  |  | 0.68 (0.30-1.50) | 0.370 |
| ***Sunlight exposure, hours / day*** | 1674 | 562 |  |  |
| ≤ 1 VS > 1 |  |  | 1.30 (0.99-1.71) | **0.054** |
| > 1 VS ≤ 1 |  |  | 0.76 (0.58-1.00) | **0.054** |
| ***Type of flooring*** | 2236 | 0 |  |  |
| Dirt / lawn VS non-dirt / lawn floor |  |  | 0.57 (0.38-0.86) | **0.007*** |
| Non-dirt/ lawn VS dirt /lawn floor |  |  | 1.74 (1.16-2.60) | **0.007*** |
| ***Body condition score*** | 1930 | 306 |  |  |
| Normal weight VS underweight |  |  | 0.96 (0.73-1.26) | 0.794 |
| Normal weight VS overweight |  |  | 0.94 (0.58-1.52) | 0.806 |
| Underweight VS over weight |  |  | 0.97 (0.58-1.63) | 0.924 |
| Overweight VS underweight |  |  | 1.02 (0.61-1.71) | 0.924 |
| ***Was the puppy vaccinated 2-4 times under 1 year of age?*** | 2205 | 31 |  |  |
| Yes VS no |  |  | 1.19 (0.34-4.10) | 0.778 |
| No VS yes |  |  | 0.83 (0.24-2.88) | 0.778 |
| ***Was the puppy dewormed 2-10 times under 1 year of age?*** | 2164 | 72 |  |  |
| Yes VS no |  |  | 0.52 (0.18-1.50) | 0.232 |
| No VS yes |  |  | 1.89 (0.66-5.41) | 0.232 |

(n): number of dogs, included dogs: the number of valid answers for the corresponding question, crude effect estimates: one covariate in the model each time, cOR: crude odds ratio, CI: confidence interval, CAD: canine atopic dermatitis, bolded: P ≤ 0.2, *: P ≤ 0.05, NPMD: non-processed meat based diet, UPCD: ultra-processed carbohydrate based diet, VS: versus.
