# Supplementary material for: Identification of modifiable pre- and postnatal dietary and environmental exposures associated with owner-reported canine atopic dermatitis in Finland using a web-based questionnaire
Source: PLoS One. 2020 May 29;15(5):e0225675. doi: 10.1371/journal.pone.0225675 (PMC7259748; doi:10.1371/journal.pone.0225675)
Supplement: S3 Table — (DOCX) [file pone.0225675.s003.docx]

**S3 Table.** **Associations between pre-, neo-, early post- and late postnatal period variables and canine atopic dermatitis based on multivariate logistic regression analyses.**

| **N.** | **Covariates** | **Total population for analysis (n=2236)** | | **Adjusted effect estimates** | |
| --- | --- | --- | --- | --- | --- |
|  |  | **Included dogs (n)** | **Missing dogs (n)** | **aOR (95% CI)** | **P-value** |
| **1** | **Model 1 (Prenatal; non-modifiable)** | 789 | 1447 |  |  |
|  | ***Maternal history of CAD*** |  |  |  |  |
|  | Non-atopic VS atopic mother |  |  | 0.07 (0.04-0.15) | **<0.001** |
|  | Atopic VS non-atopic mother |  |  | 12.60 (6.30-25.19) | **<0.001** |
|  | ***Dog breed*** |  |  |  |  |
|  | Non-allergy prone VS allergy prone  breed |  |  | 0.34 (0.22-0.53) | **<0.001** |
|  | Allergy prone VS non-allergy prone  breed |  |  | 2.89 (1.86-4.47) | **<0.001** |
|  | ***Dog color*** |  |  |  |  |
|  | <50% white colored coat VS >50% |  |  | 0.52 (0.32-0.85) | **0.010** |
|  | >50% white colored coat VS <50% |  |  | 1.91 (1.17-3.12) | **0.010** |
|  | ***Dog gender*** |  |  |  |  |
|  | Female VS male |  |  | 0.64 (0.41-1.00) | 0.052 |
|  | Male VS female |  |  | 1.55 (0.99-2.42) | 0.052 |
| **2** | **Model 2 (Prenatal; modifiable)** | 1357 | 879 |  |  |
|  | ***Mother’s diet during pregnancy*** |  |  |  |  |
|  | NPMD VS UPCD |  |  | 0.31 (0.12-0.78) | **0.013** |
|  | UPCD VS NPMD |  |  | 3.21 (1.27-8.09) | **0.013** |
|  | ***Was the mother dewormed during pregnancy?*** |  |  |  |  |
|  | Yes VS no |  |  | 0.37 (0.17-0.83) | **0.015** |
|  | No VS yes |  |  | 2.64 (1.20-5.80) | **0.015** |
| **3** | **Model 3 (Neonatal; modifiable)** | 1297 | 939 |  |  |
|  | ***Mother’s diet during lactation*** |  |  |  |  |
|  | NPMD VS UPCD |  |  | 0.69 (0.37-1.26) | 0.228 |
|  | UPCD VS NPMD |  |  | 1.44 (0.79-2.64) | 0.228 |
| **4** | **Model 4 (Early postnatal; modifiable)** | 782 | 1454 |  |  |
|  | ***Puppy’s first solid diet*** |  |  |  |  |
|  | NPMD VS UPCD |  |  | 0.31 (0.12-0.79) | **0.014** |
|  | UPCD VS NPMD |  |  | 3.20 (1.26-8.13) | **0.014** |
|  | ***Sunlight exposure, hours /day*** |  |  |  |  |
|  | ≥ 1 VS not at all |  |  | 0.56 (0.35-0.90) | **0.018** |
|  | Not at all VS ≥ 1 |  |  | 1.76 (1.10-2.82) | **0.018** |
|  | ***Body condition Score*** |  |  |  |  |
|  | Normal VS abnormal weight (↑ or  ↓) |  |  | 0.66 (0.43-0.99) | **0.048** |
|  | Abnormal (↑ or ↓) VS normal  weight |  |  | 1.51 (1.00-2.27) | **0.048** |
| **5** | **Model 5 (Late postnatal; modifiable)** | 1071 | 1165 |  |  |
|  | ***Puppy diet*** |  |  |  |  |
|  | NPMD VS UPCD |  |  | 0.69 (0.47-1.03) | 0.070 |
|  | UPCD VS NPMD |  |  | 1.43 (0.97-2.12) | 0.070 |
|  | ***Was the dog born in the same family?*** |  |  |  |  |
|  | Yes VS no |  |  | 0.28 (0.12-0.67) | **0.004** |
|  | No VS yes |  |  | 3.46 (1.48-8.08) | **0.004** |
|  | ***Sunlight exposure, hours/day*** |  |  |  |  |
|  | Zero or 1 VS > 1 |  |  | 1.15 (0.82-1.60) | 0.402 |
|  | > 1 VS zero or 1 |  |  | 0.86 (0.62-1.20) | 0.402 |
|  | ***Type of flooring*** |  |  |  |  |
|  | Dirt / lawn VS non dirt / lawn floor |  |  | 0.43 (0.23-0.78) | **0.006** |
|  | Non dirt / lawn VS dirt / lawn floor |  |  | 2.31 (1.27-4.22) | **0.006** |

(n): number of dogs, included dogs: the number of valid answers for the corresponding question, adjusted effect estimates: the estimation adjusted for the included covariates in model 1 and for dog gender in model 2, 3, 4 & 5. aOR: adjusted odds ratio, CI: confidence interval, CAD: canine atopic dermatitis, bolded: P ≤ 0.05, NPMD: non-processed meat based diet, UPCD: ultra-processed carbohydrate based diet, VS: versus.
